# Arithmetic Proficiency Across Adulthood: Cognitive and Subjective Influences

**DOI:** 10.3390/ejihpe15050084

**Published:** 2025-05-15

**Authors:** Elisabeth Goettfried, Katharina Thaler, Margarete Delazer, Demis Basso, Manuela Piazza, Michael Knoflach, Laura Zamarian

**Affiliations:** 1 Department of Neurology, Medical University Innsbruck, 6020 Innsbruck, Austria; elisabeth.goettfried@i-med.ac.at (E.G.); k.thaler@i-med.ac.at (K.T.); margarete.delazer@i-med.ac.at (M.D.); michael.knoflach@i-med.ac.at (M.K.); 2 Cognitive and Educational Sciences Lab (CESLab), Faculty of Education, Free University of Bolzano-Bozen, 39042 Bressanone-Brixen, Italy; demis.basso@unibz.it; 3 Centro de Investigación en Neuropsicologia y Neurociencias Cognitivas (CINPSI Neurocog), Universidad Católica del Maule, Talca 3605, Chile; 4 Center for Mind/Brain Sciences, University of Trento, 38068 Rovereto, Italy; manuela.piazza@unitn.it

**Keywords:** mathematics, healthy participants, subjective ratings, objective performance, competence belief

## Abstract

Arithmetic competence is crucial for navigating modern society and maintaining independence. It relies on domain-general and domain-specific cognitive skills, as well as subjective factors. Given its importance, understanding how these factors shape adult arithmetic proficiency is essential. This study investigated demographic, cognitive, and subjective influences on various arithmetic skills throughout adulthood, including both younger and older individuals. In total, 134 adults aged 20–68 completed computerized tasks assessing simple calculations, exact and approximate complex calculations, and arithmetic principles, alongside neuropsychological testing and self-ratings on math anxiety, math self-concept, attitudes toward mathematics, and the frequency of engagement with numbers. The results indicate that accuracy varied by task, with approximate calculations being the most challenging. Self-ratings showed low math anxiety but moderate-to-high math self-concept, positive attitudes, and moderate engagement with numbers. Age correlated only with arithmetic principles; however, interference inhibition and engagement with numbers, not age, best predicted performance. Executive functions correlated solely with approximate calculations and arithmetic principles, while subjective measures were related to all arithmetic tasks. The regression analyses indicate strong interrelationships, particularly among calculation tasks. The findings highlight the multifaceted nature of arithmetic competence and suggest it remains stable in adulthood, with age-related declines only evident in arithmetic principles, likely due to declining executive functions.

## 1. Introduction

The ability to understand and work with numbers is fundamental at all ages and in many aspects of our daily lives, such as adjusting recipes or paying at the grocery store. Arithmetic performance is closely related to a range of cognitive abilities that include both domain-general functions and domain-specific skills (Knops et al., 2017; LeFevre et al., 2010). Three basic types of knowledge have been identified as crucial for arithmetic (Delazer, 2003). Arithmetic fact knowledge allows for the quick recall of solutions to simple calculations directly from memory (e.g., “9 × 4 = 36”). Procedural knowledge involves applying computational procedures and algorithms to solve problems, primarily for familiar tasks (e.g., knowing how to multiply “36 × 58”). Conceptual knowledge fosters a deeper understanding of mathematical principles and numerical relationships, such as grasping the inverse relationship between addition and subtraction (Delazer, 2003).

Arithmetic competence relies heavily on various domain-general cognitive functions, including attention (LeFevre et al., 2010), language (Chow & Ekholm, 2019), spatial skills (Hawes et al., 2022), and executive functions (Bull & Lee, 2014; Cragg et al., 2017). Among these executive functions, working memory has been found to be strongly related to arithmetic performance, whether in single-digit or multi-digit arithmetic (Chen & Bailey, 2021; Raghubar et al., 2010). Set-shifting is thought to be essential for enabling individuals to switch between different operations, such as solving addition and multiplication problems alternately (Zamarian et al., 2007b), or between different problem-solving strategies (Bull & Lee, 2014; Cragg et al., 2017). Inhibition is considered to play a significant role in mental arithmetic, although the evidence regarding its effect is mixed (Bull & Lee, 2014; Gilmore et al., 2015). Inhibition appears to influence conceptual knowledge in adults but procedural competence in children, likely due to the different mechanisms involved in mental arithmetic at varying ages (Gilmore et al., 2015). It helps individuals shift their attention from procedural solutions to identifying underlying numerical relationships (Gilmore et al., 2015).

Some domain-general functions, such as executive functions, are found to decline over the lifespan (Spreng & Turner, 2019). Consequently, it may be assumed that certain arithmetic skills—particularly complex mental calculation that requires quick processing, inhibition, shifting, or the updating of information in working memory—also decline with advanced age. Research demonstrates that, compared to young adults, older adults employ fewer and less efficient strategies, compute more slowly, and are less accurate, especially as task complexity increases (Duverne & Lemaire, 2005; Hinault & Lemaire, 2016; Salthouse & Coon, 1994). This decline is thought to be associated with reduced executive control, which makes it more difficult for older adults, for example, to switch between problem-solving strategies or to inhibit interfering information (Hinault & Lemaire, 2016; Hodzik & Lemaire, 2011). In contrast, older adults appear to retain highly automated processes, such as arithmetic fact retrieval, at levels comparable to or even better than those of young adults (Zamarian et al., 2018). However, slower peripheral processing may hinder the response process in older adults, contributing to the age-related differences observed in fact retrieval speed (Zamarian et al., 2007c).

Mathematical performance may be influenced not only by cognitive factors but also by subjective factors (Dowker et al., 2016; Hernández De La Hera et al., 2023; Rossi et al., 2022; Rossi et al., 2023). Math anxiety is a psychological condition characterized by feelings of tension, apprehension, or even fear when dealing with numbers and math-related situations (Ashcraft, 2002). Symptoms can manifest physically (e.g., nervousness and physiological reactions) and cognitively (e.g., worry and self-critical thoughts; Liebert & Morris, 1967). Math anxiety can affect people of all ages (Dowker et al., 2016; Dowker & Sheridan, 2022; Hart & Ganley, 2019). Meta-analyses, primarily focusing on studies involving children, indicate a small-to-moderate negative association between math anxiety and mathematical achievement, suggesting that higher math anxiety levels are generally linked to lower mathematical performance (Barroso et al., 2021). Significant gender effects have been observed, with women typically reporting higher math anxiety levels and rating their abilities lower compared to men, although actual performance differences in gender-equitable educational settings tend to be minimal or absent (Devine et al., 2012; Dowker et al., 2016; Spelke, 2005). Mathematical performance is associated not only with math anxiety but also with attitudes toward mathematics—encompassing a range of components such as an individual’s feelings, beliefs, and opinions regarding mathematics that influence engagement with the subject—and math self-concept, which refers to one’s perception of their own mathematical competence (Di Martino & Zan, 2010; Lipnevich et al., 2016; Rossi et al., 2022; Hernández De La Hera et al., 2023). Various factors may influence math anxiety, attitudes toward mathematics, and math self-concept, including an individual’s past experiences, social factors (such as parental attitudes or peer influences), or cultural stereotypes (Rossi et al., 2022). To our knowledge, few studies (e.g., Hart & Ganley, 2019) have examined the relationship between mathematics and these subjective factors across adulthood, encompassing both younger and older individuals, rather than focusing exclusively on children or university students.

Our study aimed to fill this gap by systematically investigating basic arithmetic competence throughout adulthood, including both younger and older adults, while examining the relative contributions of different domain-general and domain-specific cognitive abilities, as well as subjective factors. Specifically, we focused on exact simple and complex computations, approximate complex computations, and the understanding of arithmetic principles as proxies of arithmetic fact knowledge, procedural knowledge, and conceptual knowledge. We were interested in (1) the effects of demographic variables (age, education, and sex); (2) the effects of domain-general cognitive functions (particularly, processing speed and executive functions); (3) the impact of subjective factors (like math anxiety, math self-concept, attitudes toward mathematics, and the frequency of engagement with numbers in daily life, educational settings, and professional contexts in the last 10 years); and (4) the predictors of arithmetic processing, which include different objective and subjective arithmetic measures, alongside demographic variables and domain-general cognitive factors. For objective arithmetic measures, we used accuracy rates from computerized tasks assessing various arithmetic skills. For subjective arithmetic measures, we employed self-rated scales adapted for adults, assessing math anxiety, math self-concept, attitudes toward mathematics, and the frequency of engagement with numbers.

We predicted that (1) older age and lower education levels would be associated with poorer arithmetic performance, particularly in more complex tasks. Whether there are differences between women and men on any of the tasks remains an open question. Furthermore, we expected that (2) better domain-general cognitive functions would be associated with better arithmetic performance. In particular, executive functions should play a key role in complex mental calculation, whether exact or approximate, and in the conceptual task. Additionally, we predicted that (3) lower math anxiety levels, more positive attitudes toward mathematics, a stronger math self-concept, and a greater frequency of engagement with numbers would be associated with better arithmetic performance. Finally, we assumed (4) close interrelationships among the different components of arithmetic. Although the different components are, in principle, independent, a highly proficient individual is likely to excel across all arithmetic tasks. Since simple facts appear to underpin proficiency in more complex computations, we expected them to serve as a significant predictor for other tasks.

## 2. Materials and Methods

### 2.1. Participants

Between December 2022 and August 2024, 134 participants were recruited through personal acquaintances and public advertisements. The inclusion criteria were being older than 18 years old, having completed at least 8 years of education, and having achieved a score of at least 27 on the Mini-Mental State Examination (MMSE) for people older than 60 years (Folstein et al., 1975). The exclusion criteria were having a history of neurological diseases (e.g., dementia), psychiatric disorders (e.g., major depression), addiction, substance abuse, or repeated surgery with general anesthesia in the past two years.

Overall, the mean age of our sample was 43.1 years (SD 14.2, range 20–68) and the mean length of education was 13.4 years (SD 3.0, range 8–21). In total, 67 participants (50%) were female. All participants were German-speaking, living in Austria or Germany, and had an estimated verbal intelligence quotient of at least 85 (Lehrl, 2005). Detailed information on the participants’ demographic characteristics by decade, as well as their performance on neuropsychological tests, arithmetic tasks, and subjective measures, can be found in Table S1 in Supplementary Materials.

With a sample size of 134 participants, we aimed to ensure sufficient statistical power to detect effect sizes (f2) between 0.10 and 0.15 (Cohen, 1988) in multiple regression models that could include up to 13 predictors (two-sided, α = 0.05, power = 0.95; G*Power version 3.1.9.7; Faul et al., 2007).

### 2.2. Neuropsychological Background Assessment

Participants completed a comprehensive neuropsychological assessment to ensure the integrity of their cognitive functioning (for a description, see Supplementary Materials). This included standardized cognitive tests of verbal episodic memory, verbal attention span, executive functions (verbal working memory, verbal fluency, set-shifting, and interference inhibition), and information processing speed (Aschenbrenner et al., 2000; Bäumler, 1985; Härting et al., 2000; Petermann et al., 2016; Smith, 1973). Additionally, a mental health questionnaire was administered (Herrmann et al., 1995). For our primary analyses, we focused specifically on verbal working memory, verbal fluency, set-shifting, interference inhibition, and information processing speed.

### 2.3. Objective Arithmetic Measures

Arithmetic competence was assessed using selected items from the Number Processing and Calculation (NPC) battery (Delazer et al., 2003) in a computerized format with E-Prime 3.0 Software (for a detailed description, see Supplementary Materials). The tasks included simple facts (FACTS, 40 items), exact complex calculations (COMPL, 40 items), approximate complex calculations (APPROX, 16 items), and arithmetic principles (PRINC, 20 items). FACTS and COMPL involved production tasks, while APPROX was a multiple-choice task requiring participants to choose the closest alternative to the correct solution. PRINC required solving problems by inference without direct calculation. While FACTS, COMPL, and APPROX assessed all four basic operations, PRINC focused specifically on addition and multiplication. In each task, the items were displayed at the center of the computer screen until a response was given or the time limit expired. The participants were instructed to enter their responses as quickly and accurately as possible. The response times (RTs) and accuracy were recorded. Analyses were conducted using the overall scores for each task, without distinguishing between operations. To facilitate more accurate comparisons between tasks with differing demands (e.g., production vs. verification), our analyses focused on accuracy rates. In this study, we primarily used RTs for quality control purposes (e.g., to identify responses that were implausibly fast in the approximation task).

### 2.4. Subjective Arithmetic Measures

We assessed math anxiety, math self-concept, attitudes toward mathematics, and the frequency of engagement with numbers using Likert-scale questionnaires that were translated and adapted for adult participants (for a description, see Supplementary Materials). Math anxiety (affective and cognitive) and math self-concept were measured using selected items from Henschel and Roick’s (2017) scale, with higher scores indicating greater anxiety or higher perceived competence. Higher scores on the Attitudes Toward Mathematics Instrument (ATMI; Tapia, 1996) reflect more positive attitudes. Finally, the participants were asked to respond to two questions regarding their frequency of interactions with numbers in everyday life, educational settings, and professional situations. Analyses were conducted on the individual median scores for each (sub)scale.

### 2.5. Procedure

Testing was conducted either at the Medical University of Innsbruck or in a quiet environment of the participant’s choice (e.g., the participant’s home or individual study rooms). Neuropsychological tests were administered in a pseudo-randomized order, followed by questionnaires on subjective arithmetic measures. The computerized arithmetic tasks were administered last to prevent any influence on responses to the subjective scales. The entire testing session lasted 75–90 min, with breaks allowed as needed.

### 2.6. Statistical Analysis

Statistical analyses were carried out with SPSS Version 29.0 (IBM, Chicago, IL, USA), employing parametric statistical methods. Separate Pearson correlation analyses were conducted to investigate the relationships between objective arithmetic performance and demographic variables, domain-general cognitive factors, and subjective arithmetic measures. The false discovery rate (FDR) method was applied to correct for multiple correlations. We conducted a series of hierarchical regression analyses with each objective arithmetic measure as the dependent variable in separate models, while including the remaining objective arithmetic measures as domain-specific regressors. Independent variables were entered into each model in the following order: first, demographic variables; second, domain-general cognitive factors; third, domain-specific variables; and finally, subjective arithmetic measures. Only variables that showed significant correlations (after FDR correction) with each arithmetic task were included in the regression analysis. The significance threshold was set at α = 0.05.

## 3. Results

### 3.1. Outcomes in Neuropsychological Background Tests

In general, group scores in neuropsychological tests were in the average range of standardized norms (for detailed information, see Supplementary Materials).

### 3.2. Objective Arithmetic Outcomes

The results are reported in Table 1. Pairwise *t*-tests indicated that accuracy rates were significantly higher when solving FACTS compared to COMPL (t(133) = 12.45, *p* < 0.001), to APPROX (t(133) = −19.26, *p* < 0.001), and to PRINC (t(133) = 10.54, *p* < 0.001). Additionally, accuracy rates in COMPL were higher than those for PRINC (t(133) = 2.12, *p* = 0.036) and for APPROX (t(133) = −11.98, *p* < 0.001). The comparison between PRINC and APPROX was also significant (t(133) = −8.95, *p* < 0.001), with APPROX being the least accurate. Ceiling performance was achieved by 7.5% (*n* = 10) of the participants in FACTS, 2.2% (*n* = 3) in COMPL, and 3.7% (*n* = 5) in PRINC. None of the participants reached ceiling performance in APPROX. Only a few participants performed below 1 SD from the group mean on each task—FACTS 14.2% (*n* = 19), COMPL 14.9% (*n* = 20), APPROX 17.2% (*n* = 23), and PRINC 10.4% (*n* = 14).

### 3.3. Subjective Arithmetic Outcomes

In general, the group’s mean scores indicated low levels of math anxiety, along with moderate-to-high levels of math self-concept, positive attitudes toward mathematics, and moderate engagement with numbers. A pairwise *t*-test showed comparable levels of affective and cognitive math anxiety (t(133) = −1.00, *p* = 0.319). Only a few participants achieved an individual median score of at least 3, suggestive of moderate-to-high levels, on the affective (*n* = 5, 3.7%) and cognitive math anxiety (*n* = 8, 6.0%) subscales. The majority of participants scored at least 3 on the subscales assessing math self-concept (*n* = 103, 76.9%) and attitudes toward mathematics (*n* = 123, 91.8%).

### 3.4. Correlation Analysis

#### 3.4.1. Correlations with Demographic Variables

There was a significant negative correlation between age and accuracy in PRINC (r = −0.295, *p* < 0.001). Education correlated positively and significantly with accuracy across all arithmetic tasks—FACTS (r = 0.312, *p* < 0.001), COMPL (r = 0.407, *p* < 0.001), APPROX (r = 0.321, *p* < 0.001), and PRINC (r = 0.201, *p* = 0.020). Sex correlated significantly with accuracy in COMPL (r = 0.228, *p* = 0.008), with women scoring lower than men. Other correlations were not significant (all *p* > 0.05).

For correlations between demographic variables and subjective arithmetic measures, please refer to Figure S1 in Supplementary Materials.

#### 3.4.2. Correlations with Domain-General Cognitive Factors

**Figure 1:**
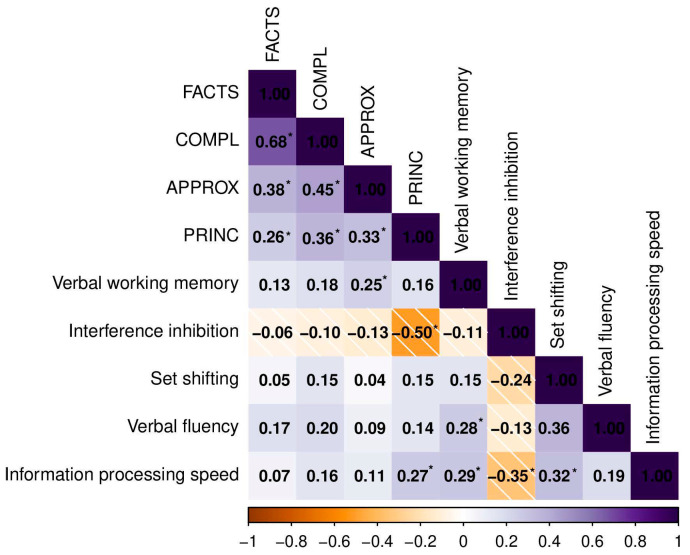
Coefficients of a Pearson correlation analysis between objective arithmetic measures and domain-general cognitive outcomes (N = 134). Notes: (*) indicates statistical significance after FDR correction; FACTS = simple arithmetic facts; COMPL = complex exact calculation; APPROX = complex approximate calculation; PRINC = arithmetic principles.

The results are presented in Figure 1. We found no significant correlations for FACTS and COMPL. In contrast, higher accuracy in APPROX significantly correlated with higher scores in a verbal working memory test. Additionally, better scores on tests of interference inhibition and information processing speed were associated with higher accuracy in PRINC.

**Figure 2:**
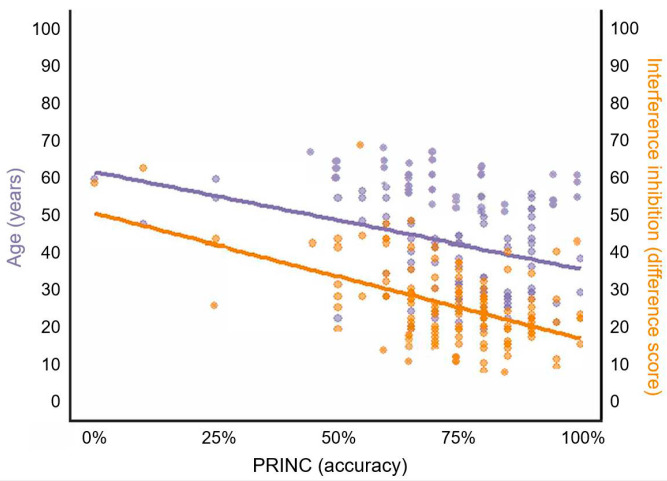
Correlation of accuracy rates in PRINC with age (in years) and interference inhibition (measured as the difference score between two Stroop sub-tests). Notes: PRINC = arithmetic principles. The best-fit line for the correlation between PRINC accuracy and age is shown in blue, while the best-fit line for the correlation between PRINC accuracy and interference inhibition is shown in orange.

Figure 2 shows the correlation of performance on the PRINC task with age (in years) and interference inhibition (measured as the difference score between two Stroop sub-tests; higher difference scores mean lower performance).

#### 3.4.3. Correlations with Subjective Arithmetic Outcomes

**Figure 3:**
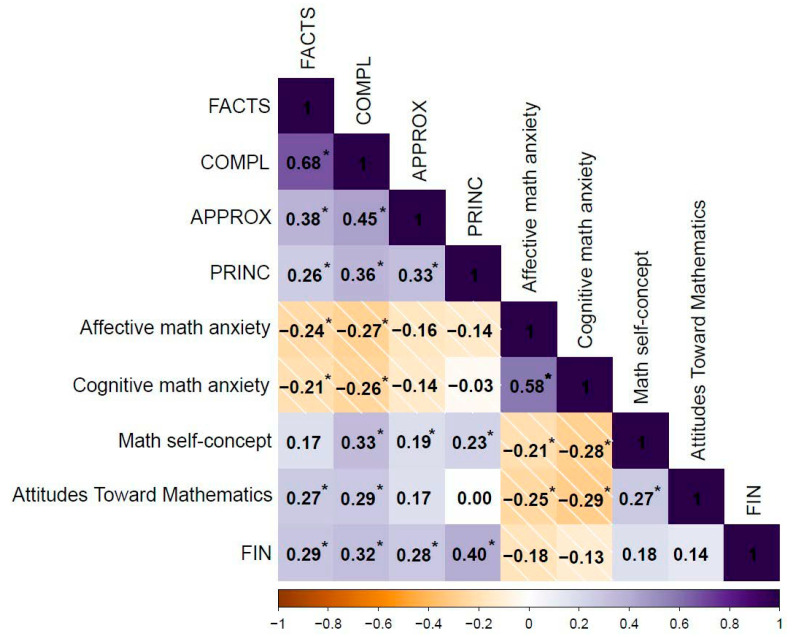
Coefficients of a Pearson correlation analysis between objective and subjective arithmetic measures (N = 134). Notes: (*) indicates statistical significance after FDR correction; FACTS = simple arithmetic facts; COMPL = complex exact calculation; APPROX = complex approximate calculation; PRINC = arithmetic principles; FIN = frequency of interactions with numbers.

The results are presented in Figure 3. Higher accuracy rates in FACTS and COMPL were associated with a greater frequency of interactions with numbers, lower anxiety levels, a more positive attitude toward mathematics, and, specifically for COMPL, a stronger math self-concept. Higher accuracy rates in APPROX and PRINC were significantly correlated with a greater frequency of interactions with numbers and, specifically for PRINC, with a stronger math self-concept. Other correlations were not significant (all *p* > 0.05).

### 3.5. Hierarchical Regression Analysis

#### 3.5.1. Simple Calculations

The results are reported in Table 2 (upper panel). The analysis conducted for FACTS showed that Model 1, which included education as a predictor, was significant (F(1, 132) = 14.22, *p* < 0.001), accounting for 9.0% of the variance. Model 2, which added objective performance in other arithmetic tasks to the predictor of Model 1, explained significantly more variance (ΔR^2^ = 0.372, F(3, 129) = 30.10, *p* < 0.001). This model accounted for 45.2% of the variance and was significant (F(4, 129) = 28.48, *p* < 0.001), with COMPL emerging as a significant predictor. Model 3, which added subjective arithmetic measures to the factors of Model 2, did not yield a significant increase in explained variance (ΔR^2^ = 0.018, F(5, 124) = 0.84, *p* = 0.520). This model accounted for 44.9% of the variance and was significant (F(9, 124) = 13.05, *p* < 0.001), with COMPL being the only significant predictor.

#### 3.5.2. Exact Complex Calculations

The results are reported in Table 2 (lower panel). In the analysis computed for COMPL, Model 1 was significant (F(2, 131) = 20.18, *p* < 0.001), explaining 22.4% of the variance. Both education and sex were identified as significant predictors. In Model 2, objective performance in other arithmetic tasks was added to the predictors of Model 1, resulting in a significant increase in explained variance (ΔR^2^ = 0.328, F(3, 128) = 32.15, *p* < 0.001). Model 2 explained 54.7% of the variance and was significant (F(5, 128) = 33.12, *p* < 0.001). The significant predictors in Model 2 were education, sex, FACTS, and PRINC. Model 3, which finally added subjective arithmetic factors to the predictors of Model 2, did not explain significantly more variance (ΔR^2^ = 0.019, F(5, 123) = 1.15, *p* = 0.339). This model accounted for 55.0% of the variance and was significant (F(10, 123) = 17.23, *p* < 0.001), with education and FACTS identified as the significant predictors.

#### 3.5.3. Approximate Complex Calculations

The results are reported in Table 3 (upper panel). The analysis of APPROX showed that Model 1, which included education as a predictor, was significant (F(1, 132) = 15.15, *p* < 0.001) and explained 9.6% of the variance. Model 2, which added verbal working memory as a predictor, significantly improved the model fit (ΔR^2^ = 0.036, F(1, 131) = 5.45, *p* = 0.021). Overall, Model 2 accounted for 12.6% of the variance and was significant (F(2, 131) = 10.56, *p* < 0.001), with both education and verbal working memory identified as significant predictors. In Model 3, objective performance in other arithmetic tasks was added to the predictors of Model 2, resulting in a significant increase in explained variance (ΔR^2^ = 0.138, F(3, 128) = 8.14, *p* < 0.001). This model was significant (F(5, 128) = 9.80, *p* < 0.001) and accounted for 24.9% of the variance, with COMPL and PRINC emerging as significant predictors. Model 4, which added a subjective arithmetic measure to the predictors of Model 3, did not account for a significant increase in variance (ΔR^2^ = 0.006, F(1, 127) = 1.05, *p* = 0.307). Overall, Model 4 explained 24.9% of the variance and was significant (F(6, 127) = 8.34, *p* < 0.001), with COMPL being the only significant predictor.

#### 3.5.4. Arithmetic Principles

The results are reported in Table 3 (lower panel). In the analysis conducted for PRINC, Model 1 was significant (F(2, 131) = 9.08, *p* < 0.001), accounting for 10.8% of the variance, with both age and education as significant predictors. Model 2 improved upon Model 1, accounting for significantly more variance (ΔR^2^ = 0.167, F(2, 129) = 15.15, *p* < 0.001). Model 2 explained 26.7% of the variance and was significant (F(4, 129) = 13.10, *p* < 0.001), with interference inhibition emerging as the only significant predictor. Model 3, which added objective performance in other arithmetic tasks to the predictors of Model 2, accounted for a further significant increase in variance (ΔR^2^ = 0.100, F(3, 126) = 6.84, *p* < 0.001). Model 3 explained 35.4% of the variance and was significant (F(7, 126) = 11.43, *p* < 0.001), with interference inhibition and COMPL emerging as significant predictors. Finally, Model 4, which added subjective arithmetic measures to the predictors of Model 3, also accounted for a significant increase in variance (ΔR^2^ = 0.063, F(2, 124) = 7.06, *p* < 0.001). Overall, Model 4 explained 41.1% of the variance and was significant (F(9, 124) = 11.31, *p* < 0.001). The significant predictors identified in this model were interference inhibition and frequency of interactions with numbers.

In all analyses, the Variance Inflation Factor (VIF) indicated that multicollinearity was not a concern. In summary, the hierarchical regression analyses indicated significant model improvements, particularly when adding competence in other arithmetic tasks as predictors.

## 4. Discussion

This study investigated basic arithmetic competence throughout adulthood, encompassing both younger and older adults, while evaluating how domain-general cognitive functions, domain-specific skills, and subjective arithmetic factors contribute to performance. Overall, we found that the accuracy rate varied across tasks, with simple computations yielding the most accurate responses (mean 89% correct), while approximate complex computations resulted in the least accurate answers (mean 57% correct). These findings align with previous studies on various aspects of arithmetic competence in healthy adults (Delazer et al., 2003). The results from the subjective arithmetic scales indicated that levels of math anxiety were low in our sample, with means of 1.3 on a Likert scale from 1 (“does not apply at all”) to 4 (“fully applies”), especially when compared to those reported for younger samples (e.g., elementary school children, with means of 2.0 and 2.2 on the cognitive and affective math anxiety subscales used in our study; Henschel & Roick, 2017). In general, math self-concept, attitudes toward mathematics, and the frequency of handling numbers in daily life, educational settings, and work contexts were rated as moderate-to-high. We will discuss our results in the same order as we made our aims and predictions.

### 4.1. Demographic Factors

An age effect was found only in the arithmetic principles task, which assessed conceptual knowledge. Other arithmetic tasks showed no decline in accuracy with increasing age. Contrary to expectations, performance on both exact and approximate complex calculations was not negatively associated with age. Notably, our study focused on accuracy rather than response time, which prior research has shown to decrease with age (Avcil & Artemenko, 2025; Hinault & Lemaire, 2016; Salthouse & Coon, 1994; Zamarian et al., 2018). Similarly, a recent cross-sectional study on number processing and calculation skills (Avcil & Artemenko, 2025) found comparable accuracy in complex addition and subtraction tasks among younger, middle-aged, and older adults. In our study, we also found that higher arithmetic competence was associated with higher education levels, aligning with expectations and prior studies (OECD, 2024; Ritchie & Bates, 2013). Our observation that men outperformed women in only one task—namely, exact complex calculations—is consistent with research suggesting minimal gender differences in arithmetic performance (e.g., Rossi et al., 2023). In countries promoting gender equality in education, differences in number processing and arithmetic skills are often negligible or absent (Guiso et al., 2008), supporting the view that such disparities mostly stem from social factors (e.g., educational opportunities or societal role stereotypes) rather than innate ability (Hyde & Mertz, 2009; Spelke, 2005).

### 4.2. Domain-General Cognitive Factors

Our findings reveal that domain-general cognitive factors are more strongly associated with “non-exact” arithmetic performance—namely, approximate complex calculations and understanding of arithmetic principles—than with “exact” arithmetic performance. Specifically, performance on simple computations, which serves as a proxy for arithmetic fact knowledge, and on exact complex calculations, was not linked to any domain-general cognitive functions. In contrast, better performance on approximate complex computations was related to higher verbal working memory capacity, while increased accuracy on the arithmetic principles task was associated with faster information processing and stronger inhibitory control. These results suggest that, in adults, domain-general factors play a more relevant role in supporting arithmetic performance on tasks involving approximation or conceptual understanding, rather than on those relying on the retrieval of arithmetic facts from memory or exact calculations. Existing research supports this, indicating that cognitive functions such as working memory, inhibition, and set-shifting are particularly important when arithmetic skills are less automatized, newly learned, or cognitively demanding (Desoete, 2015; Hinault & Lemaire, 2016; Zamarian et al., 2007b; Zamarian et al., 2018). Our study aligns with this research, showing no significant correlations with domain-general cognitive factors for exact (simple and complex) calculations, while the significant associations observed for approximate calculations and arithmetic principles underscore the greater relevance of domain-general cognitive factors for “non-exact” arithmetic performance.

### 4.3. Subjective Arithmetic Factors

Our study reveals several important findings regarding subjective arithmetic factors in adulthood. First, we observed that higher arithmetic competence was associated with lower math anxiety levels, more positive attitudes toward mathematics, a stronger math self-concept, and a greater frequency of using numbers in daily life, educational settings, and professional contexts. Second, our results indicate that math anxiety levels, particularly on the affective scale, tend to decrease with age (see Figure S1 in Supplementary Materials).

These findings build on prior research (Dowker et al., 2016; Hernández De La Hera et al., 2023; Rossi et al., 2022, 2023) by demonstrating that different dimensions of basic arithmetic competence—including simple computations, exact and approximate complex computations, and understanding of arithmetic principles—relate to various subjective arithmetic factors. Specifically, the observed associations between arithmetic skills and math anxiety, math self-concept, and attitudes toward mathematics highlight the interplay between objective and subjective arithmetic components across adulthood.

While much research has examined math anxiety in children, adolescents, and university students (Barroso et al., 2021), few studies have investigated it in the general adult population (Hart & Ganley, 2019) or older individuals (Donelle et al., 2007). Our findings contribute to filling this gap by showing decreasing math anxiety with age, which aligns with aging research suggesting changes in emotion regulation—particularly, a greater focus on positive information, goals, and experiences—in later life (Mather, 2016; Mather & Carstensen, 2005).

### 4.4. Predictors of Arithmetic Performance

The regression analyses that included demographic variables and domain-general cognitive factors, alongside objective and subjective arithmetic measures, explained a total of 45%, 55%, 25%, and 41% of the variance in simple computations, exact complex computations, approximate complex computations, and arithmetic principles. Several aspects of these analyses are particularly noteworthy. First, the inclusion of proficiency in other arithmetic tasks in the regression models accounted for an additional 37% of the variance in simple computations, 33% in exact complex computations, 14% in approximate complex computations, and 10% in the arithmetic principles task. These contributions, especially in computation tasks, were greater than those attributable to demographic variables, domain-general cognitive factors, and subjective arithmetic outcomes. Second, proficiency in other arithmetic tasks emerged as a significant predictor of performance on both simple and complex computations (whether exact or approximate), but not for the arithmetic principles task. Specifically, performance on exact complex computations was identified as a significant predictor of performance on both simple computations and approximate complex computations, while proficiency in simple computations emerged as a significant predictor of performance on exact complex computations. Proficiency in exact complex computations clearly provides an advantage when solving simple calculations or estimating solutions to complex arithmetic problems. Since exact complex calculations can be approached by breaking the problem down into smaller components (Zamarian et al., 2007a), proficiency in retrieving arithmetic facts aids in performing complex arithmetic problems.

Notably, while objective arithmetic measures predicted performance across calculation tasks, subjective measures showed predictive value only for arithmetic principles. After controlling for other factors, the strongest predictors of performance in the arithmetic principles task were interference inhibition and self-reported engagement with numbers in daily life, educational settings, and professional contexts. None of the other arithmetic tasks emerged as significant predictors, consistent with neuropsychological findings from single-case studies of brain-damaged patients, which demonstrates dissociations among factual, procedural, and conceptual knowledge (for reviews, Delazer, 2003; Zamarian et al., 2007a). These results suggest that, in healthy adults, competence with arithmetic principles (e.g., commutativity, associativity, and distributivity) relies on distinct mechanisms from proficiency in computation, regardless of whether calculations are exact, approximate, simple, or complex. Understanding and applying arithmetic principles reflect arithmetic conceptual knowledge (Delazer, 2003). Regular engagement with numbers, alongside inhibitory control (see also Gilmore et al., 2015), may help adults develop greater automaticity in recognizing numerical patterns and relationships while strengthening their conceptual understanding of arithmetic principles. General inductive reasoning abilities might also contribute to the comprehension and application of mathematical concepts (Christou & Papageorgiou, 2007). While our findings highlight relationships between arithmetic conceptual knowledge, executive functions, and real-world numerical experiences, future research should directly examine how inductive reasoning influences performance on arithmetic principles tasks across adulthood.

### 4.5. Limitations and Strengths

We ought to highlight some limitations. The results, particularly concerning the subjective arithmetic measures, may be somewhat biased, as it is possible that only individuals with positive attitudes toward mathematics chose to participate. Future investigations involving adults should aim to increase variability by including individuals with high levels of math anxiety, low math self-concept, and negative attitudes toward mathematics. This approach could help clarify the distinct contributions of various subjective factors—beyond the frequency of engagement with numbers in daily life—to the comprehension and application of arithmetic principles across adulthood. Additionally, it may lead to a deeper understanding of how subjective factors influence the relationship between domain-general cognitive functions and arithmetic performance in older adults. As suggested by Zhang et al. (2018), the contribution of executive functions may become particularly evident in individuals with high levels of math anxiety, which can deplete the cognitive resources necessary for excelling in arithmetic. Furthermore, we assessed subjective arithmetic factors using shortened versions of existing measures, which were adapted for a general adult population. Future studies should evaluate the reliability of these adapted scales. Additionally, our study design does not allow for a detailed investigation of the direction of causation between objective and subjective arithmetic outcomes. While research indicates that math anxiety may significantly influence an individual’s math self-concept in both children and university students (Justicia-Galiano et al., 2017; Rossi et al., 2022), the relationship between math anxiety and mathematical performance is likely bidirectional (Carey et al., 2016), complicating the analysis in cross-sectional studies such as ours. A key strength of our study lies in being the first to investigate the relative contributions of various domain-general cognitive functions, domain-specific skills, and subjective factors to basic arithmetic competence across adulthood. Additionally, our inclusion of a broad adult age range (20–68 years) enhances generalizability compared to the university student samples typically used in prior research. This approach yields more representative data on adult cognitive profiles by encompassing both younger and older individuals.

## 5. Conclusions

Competence in performing exact mental calculations (whether simple or complex), estimating approximate solutions to complex arithmetic problems, and applying arithmetic principles (e.g., the commutative property) constitutes a fundamental goal of basic education. This competence remains essential throughout adulthood for maintaining independent functioning. Research has consistently shown that individuals with low numeracy skills face significant disadvantages compared to those with high numeracy (Reyna et al., 2009). Our study demonstrates that most arithmetic abilities remain largely stable across adulthood and are associated with positive feelings, beliefs, and attitudes toward mathematics. Arithmetic encompasses several independent components that tend to remain stable with aging, although performance on arithmetic principles presents an important exception. While advanced age shows minimal effects on factual and procedural knowledge, it may negatively impact arithmetic conceptual knowledge. Our findings reveal a strong correlation between arithmetic principles and interference inhibition, which is known to decline with age (Spreng & Turner, 2019). Beyond interference inhibition, we found that the frequency of engagement with numbers in everyday situations significantly predicts performance on the arithmetic principles task. This finding reinforces the importance of including subjective measures when testing arithmetic proficiency in the general adult population. Notably, higher objective arithmetic competence is associated not only with more positive feelings toward mathematics but also with greater engagement with numbers in daily activities.

## Figures and Tables

**Table 1:** Scores in objective and subjective arithmetic measures (N = 134).

	Max. Score	M	SD	Min	Max
FACTS (% correct)	100	88.9	9.8	57.5	100.0
COMPL (% correct)	100	76.7	15.1	22.5	100.0
APPROX (% correct)	100	56.8	20.5	0.0	91.7
PRINC (% correct)	100	73.5	16.2	0.0	100.0
Affective math anxiety (individual median)	4	1.3	0.5	1	3
Cognitive math anxiety (individual median)	4	1.3	0.5	1	3
Math self-concept (individual median)	4	2.8	0.7	1	4
Attitudes Toward Mathematics (individual median)	4	3.3	0.6	2	4
FIN (individual median)	10	6.1	2.2	1	10

Notes: M = mean; SD = standard deviation; Min = minimum; Max = maximum; FACTS = simple calculation; COMPL = exact complex calculation; APPROX = approximate complex calculation; PRINC = arithmetic principles; FIN = frequency of interactions with numbers.

**Table 2:** Coefficients of hierarchical regression analyses where performance in objective arithmetic measures (FACTS/COMPL) is the dependent variable (N = 134).

			Unstandardized Coefficients		Standardized Coefficients			Collinearity	
	Model		B	SE	Beta (β)	t	*P*	Tolerance	VIF
FACTS	1	(Intercept)	0.749	0.037		20.198	<0.001		
		Education	0.010	0.003	0.312	3.772	**<0.001**	1.000	1.000
	2	(Intercept)	0.534	0.041		13.170	<0.001		
		Education	0.001	0.002	0.028	0.394	0.694	0.809	1.235
		COMPL	0.413	0.051	0.631	8.114	**<0.001**	0.681	1.468
		APPROX	0.040	0.036	0.084	1.128	0.261	0.749	1.336
		PRINC	0.000	0.043	0.000	−0.003	0.998	0.833	1.200
	3	(Intercept)	0.534	0.064		8.312	<0.001		
		Education	0.001	0.002	0.034	0.469	0.640	0.793	1.262
		COMPL	0.398	0.054	0.609	7.371	**<0.001**	0.608	1.646
		APPROX	0.033	0.036	0.068	0.913	0.363	0.737	1.357
		PRINC	0.001	0.046	0.001	0.015	0.988	0.719	1.391
		Affective math anxiety	−0.008	0.018	−0.037	−0.454	0.650	0.630	1.588
		Cognitive math anxiety	−0.001	0.017	−0.005	−0.058	0.954	0.607	1.646
		Math self-concept	−0.014	0.011	−0.094	−1.304	0.195	0.795	1.259
		Attitudes Toward Mathematics	0.014	0.011	0.086	1.210	0.229	0.822	1.216
		FIN	0.003	0.003	0.067	0.927	0.356	0.784	1.276
COMPL	1	(Intercept)	0.360	0.065		5.507	<0.001		
		Education	0.021	0.004	0.430	5.612	**<0.001**	0.992	1.008
		Sex (f = 1, m = 2)	0.079	0.023	0.265	3.455	**<0.001**	0.992	1.008
	2	(Intercept)	−0.264	0.086		−3.072	0.003		
		Education	0.009	0.003	0.189	2.936	**0.004**	0.820	1.219
		Sex (f = 1, m = 2)	0.038	0.018	0.127	2.091	**0.038**	0.922	1.085
		FACTS	0.796	0.100	0.521	7.943	**<0.001**	0.793	1.262
		APPROX	0.093	0.049	0.127	1.885	0.062	0.752	1.330
		PRINC	0.121	0.059	0.130	2.058	**0.042**	0.858	1.166
	3	(Intercept)	−0.254	0.110		−2.300	0.023		
		Education	0.008	0.003	0.151	2.271	**0.025**	0.769	1.300
		Sex (f = 1, m = 2)	0.025	0.019	0.085	1.321	0.189	0.826	1.211
		FACTS	0.759	0.104	0.496	7.319	**<0.001**	0.737	1.357
		APPROX	0.087	0.050	0.118	1.743	0.084	0.742	1.348
		PRINC	0.106	0.063	0.114	1.675	0.096	0.735	1.361
		Affective math anxiety	−0.005	0.025	−0.017	−0.222	0.824	0.612	1.633
		Cognitive math anxiety	−0.015	0.023	−0.047	−0.629	0.531	0.606	1.651
		Math self-concept	0.010	0.016	0.039	0.598	0.551	0.809	1.236
		Attitudes Toward Mathematics	0.024	0.015	0.105	1.586	0.115	0.772	1.295
		FIN	0.002	0.005	0.027	0.408	0.684	0.777	1.287

Notes: FACTS = simple calculation; COMPL = exact complex calculation; APPROX = approximate complex calculation; PRINC = arithmetic principles; FIN = frequency of interactions with numbers. The significant statistical results (*p* < 0.050) are highlighted in bold.

**Table 3:** Coefficients of hierarchical regression analyses where performance in objective arithmetic measures (APPROX/PRINC) is the dependent variable (N = 134).

			Unstandardized Coefficients		Standardized Coefficients			Collinearity	
	Model		B	SE	Beta (β)	t	*p*	Tolerance	VIF
APPROX	1	(Intercept)	0.276	0.077		3.584	<0.001		
		Education	0.022	0.006	0.321	3.893	**<0.001**	1.000	1.000
	2	(Intercept)	0.168	0.089		1.893	0.061		
		Education	0.019	0.006	0.285	3.450	**<0.001**	0.965	1.036
		Verbal working memory	0.021	0.009	0.193	2.335	**0.021**	0.965	1.036
	3	(Intercept)	−0.259	0.154		−1.683	0.095		
		Education	0.009	0.006	0.132	1.583	0.116	0.816	1.225
		Verbal working memory	0.015	0.008	0.139	1.790	0.076	0.943	1.061
		FACTS	0.239	0.214	0.115	1.120	0.265	0.536	1.865
		COMPL	0.317	0.150	0.233	2.114	**0.036**	0.466	2.144
		PRINC	0.205	0.103	0.162	1.997	**0.048**	0.855	1.170
	4	(Intercept)	−0.249	0.154		−1.616	0.108		
		Education	0.009	0.006	0.131	1.578	0.117	0.816	1.225
		Verbal working memory	0.014	0.008	0.133	1.718	0.088	0.939	1.065
		FACTS	0.217	0.215	0.104	1.011	0.314	0.531	1.884
		COMPL	0.304	0.150	0.223	2.022	**0.045**	0.463	2.159
		PRINC	0.170	0.108	0.135	1.576	0.118	0.771	1.297
		FIN	0.008	0.008	0.087	1.026	0.307	0.792	1.263
PRINC	1	(Intercept)	0.741	0.074		9.965	<0.001		
		Age	−0.003	0.001	−0.286	−3.484	**<0.001**	0.998	1.002
		Education	0.010	0.004	0.187	2.285	**0.024**	0.998	1.002
	2	(Intercept)	0.853	0.134		6.347	<0.001		
		Age	−0.002	0.001	−0.139	−1.594	0.113	0.723	1.383
		Education	0.007	0.004	0.123	1.612	0.109	0.948	1.055
		Interference inhibition	−0.006	0.001	−0.428	−5.300	**<0.001**	0.844	1.185
		Information processing speed	0.001	0.002	0.031	0.341	0.734	0.687	1.455
	3	(Intercept)	0.649	0.151		4.284	<0.001		
		Age	−0.002	0.001	−0.136	−1.618	0.108	0.687	1.455
		Education	−0.001	0.004	−0.025	−0.312	0.755	0.784	1.275
		Interference inhibition	−0.006	0.001	−0.415	−5.455	**<0.001**	0.840	1.191
		Information processing speed	0.000	0.001	0.011	0.125	0.900	0.683	1.465
		FACTS	0.086	0.161	0.052	0.530	0.597	0.504	1.982
		COMPL	0.238	0.111	0.221	2.147	**0.034**	0.457	2.186
		APPROX	0.117	0.063	0.148	1.845	0.067	0.758	1.320
	4	(Intercept)	0.611	0.146		4.197	<0.001		
		Age	−0.002	0.001	−0.131	−1.633	0.105	0.687	1.456
		Education	−0.002	0.004	−0.037	−0.483	0.630	0.772	1.296
		Interference inhibition	−0.006	0.001	−0.411	−5.619	**<0.001**	0.826	1.210
		Information processing speed	0.000	0.001	−0.023	−0.283	0.778	0.665	1.503
		FACTS	0.053	0.155	0.033	0.344	0.731	0.497	2.011
		COMPL	0.161	0.109	0.150	1.479	0.142	0.431	2.319
		APPROX	0.084	0.061	0.107	1.379	0.170	0.741	1.349
		Math self-concept	0.027	0.018	0.111	1.514	0.133	0.830	1.205
		FIN	0.018	0.005	0.240	3.320	**0.001**	0.850	1.177

Notes: FACTS = simple calculation; COMPL = exact complex calculation; APPROX = approximate complex calculation; PRINC = arithmetic principles; FIN = frequency of interactions with numbers. The significant statistical results (*p* < 0.050) are highlighted in bold.

## Data Availability

Data available at https://doi.org/10.5281/zenodo.15172386.

## Supplementary Materials

The following supporting information can be downloaded at: https://www.mdpi.com/article/10.3390/ejihpe15050084/s1, Figure S1: Coefficients of a Pearson correlation analysis between subjective arithmetic outcomes and demographical variables for the whole sample (N = 134); Table S1: Sociodemographic variables, neuropsychological test scores, objective arithmetic measures, and subjective arithmetic measures across decades; Table S2: Scores in the neuropsychological background tests that were entered in the correlation analysis for the whole sample (N = 134).
