# Supplementary material for: Arithmetic Proficiency Across Adulthood: Cognitive and Subjective Influences
Source: Eur J Investig Health Psychol Educ. 2025 May 15;15(5):84. doi: 10.3390/ejihpe15050084 (PMC12110183; doi:10.3390/ejihpe15050084)
Supplement: Supplementary file 1 [file ejihpe-15-00084-s001.zip › ejihpe-3589278-supplementary.pdf]

Supplementary Material

Detailed information about the sample

Table S1 reports detailed information about the participants' demographic characteristics by decade, as well as their performance on neuropsychological tests, objective arithmetic tasks, and subjective arithmetic measures. A short description of the neuropsychological tests, as well as objective and subjective arithmetic measures can be found below.

Table S1. Sociodemographic variables, neuropsychological test scores, objective arithmetic measures, and subjective arithmetic measures across decades.

	20-29 years (n=33)		30-39 years (n=24)		40-49 years (n=22)		50-59 years (n=33)		60-68 years (n=22)	
	M	*SD*	*M*	*SD*	*M*	*SD*	*M*	*SD*	*M*	*SD*
Sociodemographic variables										
Age	25.1	2.7	33.3	3.3	44.7	2.7	54.3	2.4	62.7	2.5
Sex (female)	16		11		12		17		11	
Education	13.4	2.0	13.8	3.0	13.4	3.6	13.4	3.2	13.1	3.6
Neuropsychological background scores										
Verbal working memory (test score)	7.1	1.8	7.2	1.7	6.7	1.5	6.5	2.1	6.5	2.2
Interference inhibition (difference score)	22.5	8.2	25.2	7.7	25.4	13.6	29.0	11.5	31.3	10.8
Set shifting (sports-fruits /min)	17.3	2.8	16.0	3.7	16.0	2.6	14.5	2.9	14.0	2.5
Verbal fluency (mean score)	21.7	5.3	21.5	3.1	22.1	4.9	23.9	5.1	21.0	4.2
Information processing speed (correct answers)	66.8	9.8	67.5	8.6	62.7	7.0	60.2	7.2	53.3	7.0
Subjective arithmetic measures										
Affective math anxiety (individual median)	1.6	0.6	1.2	0.3	1.2	0.4	1.2	0.3	1.3	0.5
Cognitive math anxiety (individual median)	1.5	0.5	1.2	0.5	1.3	0.5	1.2	0.4	1.3	0.4
Math self-concept (individual median)	2.8	0.5	3.0	0.5	3.0	0.6	2.8	0.8	2.5	0.8
Attitudes Toward Mathematics (individual median)	3.1	0.6	3.4	0.6	3.2	0.6	3.4	0.6	3.3	0.6
FIN (individual median)	6.2	1.9	6.3	2.0	5.7	2.2	6.4	2.2	5.8	2.8
Objective arithmetic measures										
FACTS (% correct)	86.9	11.3	88.4	9.0	87.6	10.3	90.6	9.4	89.2	08.8
COMPL (% correct)	78.2	15.6	76.5	15.6	77.0	14.2	76.1	15.8	75.2	14.5
APPROX (% correct)	56.4	19.2	62.8	18.2	53.8	21.9	55.8	20.8	55.3	23.2
PRINC (% correct)	77.1	10.2	79.4	10.2	71.6	17.2	74.7	16.4	61.6	21.6

Notes: M = mean; *SD* = standard deviation; FIN = Frequency of interaction with numbers; FACTS = simple calculation; COMPL = exact complex calculation; APPROX = approximate complex calculation; PRINC = arithmetic principles.

Short description of neuropsychological background tests

Tests of verbal intelligence (Lehrl, 2005) and global cognitive status (Mini-Mental State Examination – MMSE; Folstein et al., 1975) were administered at recruitment. Following recruitment, participants completed a comprehensive neuropsychological background assessment to ensure the integrity of their cognitive functioning. The assessment included tests of verbal memory (word list learning, immediate and delayed free recall, and correct recognition of the Neuropsychological Assessment Battery – NAB; Petermann et al., 2016), verbal attention span (digit span forward subtest of the Wechsler Memory Scale – WMS; Härting et al., 2000), verbal working memory (digit span backward subtest of the WMS; Härting et al., 2000), semantic verbal fluency (animals/min subtest of the Regensburger Wortflüssigkeits-Test – RWT; Aschenbrenner et al., 2000), phonemic verbal fluency (s-words/min subtest of the RWT; Aschenbrenner et al., 2000), set shifting (sports-fruits/min subtest of the RWT; Aschenbrenner et al., 2000), interference inhibition (Stroop test – difference score; Bäumler, 1985), and information processing speed (Symbol Digit Modalities Test – SDMT; Smith, 1973), as well as a questionnaire on current anxiety and depression symptoms (HADS-D; Hospital Anxiety and Depression Scale – Deutsche Version; Herrmann et al., 1995). Our analyses focused on specific neuropsychological variables**:** verbal working memory, verbal fluency, set-shifting, interference inhibition, and information processing speed.

Short description of results on neuropsychological background tests

The mean estimated verbal intelligence quotient was 111.6 (SD 13.0, range 92-145). Twenty-two participants completed the MMSE and obtained a mean score of 29.6 (SD 0.6, range 28-30). In all cognitive tests, group scores were in the average range of standardized norms. None of the participants showed evident cognitive impairments. Table S2 reports the results in the cognitive tests that were entered in the correlation analysi**s.** Verbal working memory (test score) refers to the number of correctly recalled sequences in the digit span backward sub-test of the WMS (Härting et al., 2000). Information processing speed (correct answers) reflects the total number of correct responses in the SDMT (Smith, 1973). Set shifting (sports-fruits/min) represents the number of correct words generated in the RWT sub-test within a minute (Aschenbrenner et al., 2000), where participants have to alternate between two semantic categories (sports and fruits). Verbal fluency (mean score) indicates the average number of correct words generated across two sub-tests of the RWT (animals/min and s-words/min; Aschenbrenner et al., 2000). Finally, interference inhibition (difference score) is the reaction time difference between two sub-tests of the Stroop task (Bäumler, 1985). While greater difference scores in interference inhibition indicate lower performance, higher scores in the remaining tests signify better performance.

Table S2. Scores in the neuropsychological background tests that were entered in the correlation analysis for the whole sample (N = 134).

	Max. score	*M*	*SD*	Min	Max
Verbal working memory (test score)	12	6.8	1.9	3	11
Verbal fluency (mean score)		22.1	4.7	12	41
Set shifting (sports-fruits /min)		15.6	3.1	8	28
Interference inhibition (difference score)		26.5	10.8	8	69
Information processing speed (correct answers)	110	62.4	9.4	42	91

Notes: M = mean; *SD* = standard deviation; Min = minimum; Max = maximum. No maximum value is reported for the verbal fluency and the set shifting tasks, as there is no fixed upper limit; the number of responses is theoretically "unlimited". Similarly, no maximum value is given for the interference inhibition (difference score), since it reflects the difference between two sub-test scores.

Short description of objective arithmetic measures

Items from the Number Processing and Calculation (NPC) battery were presented in a computerized format, including simple facts (FACTS, 40 items), exact complex calculations (COMPL, 40 items), approximate complex calculations (APPROX, 16 items), and arithmetic principles (PRINC, 20 items; Delazer et al., 2003). In each task, items were displayed at the center of the computer screen (black background, white font) until a response was given or time limit expired (FACTS: 5000 ms, COMPL: 10000 ms, APPROX: 8000 ms, PRINC: 8000 ms). Participants were instructed to enter their responses (numeric keyboard: FACTS, COMPL, PRINC; mouse click: APPROX) as quickly and accurately as possible. Both response times (RTs) and accuracy were recorded. FACTS included simple addition (e.g., 5+7=?, answer range: 5-14, n=10), subtraction (e.g., 14–8=?, answer range: 2-9, n=10), multiplication (e.g., 9x5=?, answer range: 12-56, n=10), and division problems (e.g., 63:7=?, answer range: 2-9, n=10). COMPL included 2d-2d addition (e.g., 28+19=?, answer range: 34-67, n=10), 2d-2d subtraction (e.g., 67–29=?, answer range: 17-43, n=10), 2d-1d multiplication (e.g., 16x3=?, answer range: 39-69, n=10), and 2d-1d division problems (e.g., 52:4=?, answer range: 12-19, n=10). The APPROX multiple-choice task (e.g., 280x3=?, alternatives: 1600, 1050, 900, 750) included 3d-3d addition (e.g., 328+657=?, alternatives range: 450-1400 [correct solution: 570-980], n=4), 3d-3d subtraction (e.g., 956–369=?, alternatives range: 170-900 [correct solution: 290-580], n=4), 3d-1d multiplication (e.g., 539x6=?, alternatives range: 750-4300 [correct solution: 900-3200], n=4), and 3d-1d division problems (e.g., 942:3=?, alternatives range: 45-360 [correct solution:140-320], n=4). In the PRINC task, participants were shown 10 pairs of multi-digit addition and 10 pairs of multiplication problems, each consisting of one solved and one unsolved problem (e.g., 56x17=952 17x56=?; answer range: 29-36000). The addition items assessed principles such as commutativity, a + 1, a - 1, 10a + 10b, and addition-subtraction inversion (with n=2 each), while the multiplication items covered principles such as commutativity, repeated addition, 10a × 10b, (a - 1) × b,

and multiplication-division inversion (with n=2 each; Delazer et al., 2003). Participants were instructed to infer the solution to the unsolved problem based on its relationship to the solved problem, without performing any direct calculations. To ensure that our participants were not performing calculations, we first explicitly emphasized in our instructions that they should rely solely on their understanding of the relationships between problems, rather than on performing calculations. We also included a practice trial before each of the two blocks, allowing participants to apply this approach and thereby reinforce the instruction. Second, we limited the time available for each problem. Third, we used problems where the solutions could be easily inferred through reasoning about the relationships between the problems. Finally, we allowed participants to verbalize their thoughts during the task, providing additional qualitative evidence that they were relying on inference rather than calculation. We also monitored performance throughout the task. In each task, the different operations (addition, subtraction, multiplication, division) were tested block-wise. However, the analyses were conducted using the overall scores for each task, without distinguishing between operations.

Short description of subjective arithmetic measures

Selected items from the Henschel and Roick's (2017) scale (category: application) were used to assess cognitive math anxiety (worry about failure: e.g., "I am worried that I don't notice getting shortchange when shopping.", n=4) and affective math anxiety (nervousness: e.g., "How nervous are you when you want to cook pudding for four people but the quantities in the recipe are only for three people?", n=4) . The competence belief scale (mathematics self-concept: e.g., "I am good at estimating my chances of winning when playing.", n=8) was used as a proxy of math self-concept and is therefore also referred to as such in the main manuscript. The Henschel and Roick's (2017) scale was originally developed to assess cognitive and affective components of math anxiety, as well as students' math self-concept, across different settings (e.g., evaluation, learning, application) and mathematical content areas (e.g., arithmetic, geometry, stochastic). In this study, we focused on items related to "application" because they are more easily adaptable for a general adult population. Higher scores on the anxiety scales indicate greater levels of anxiety, while higher scores on the math self-concept scale indicate greater perceived mathematical ability. In this study, Cronbach's alpha was 0.72 for the anxiety items and 0.83 for the math self-concept items. Ten items from the Attitudes Toward Mathematics Instrument (ATMI; Tapia, 1996) were selected to explore participants' general attitudes toward mathematics. We specifically chose items from the "perceived value of mathematics" category to focus on individuals' opinions about the usefulness, relevance, and worth of mathematics in life (e.g., "Mathematics is one of the most important subjects for people to study."). Higher scores on this scale were generally interpreted as indicating more positive attitudes toward mathematics. In this study, Cronbach's alpha for this measure was 0.83. All these questionnaires were translated from English into German by two

independent German-speaking researchers, each with C1 proficiency in English. In case of disagreement between the translations, a third independent researcher was asked to perform a back translation. Some of the items were adapted to be more appropriate for an adult population (e.g., "High school math courses would be very helpful no matter what I decide to study" was changed to "Math courses at school are very helpful no matter what you want to do professionally"). Responses were collected using a 4-point Likert scale (1: "does not apply at all", 2: "does rather not apply", 3: "partially applies", 4: "fully applies"). Finally, participants were asked to respond to two questions on frequency of interactions with numbers in educational/work settings ("How often have I had to deal with numbers in my education/work in the last ten years (e.g., mental arithmetic, calculating percentages, converting measurements, estimating quantities)?") and in everyday life ("How often have I had to deal with numbers in my everyday life in the last ten years (e.g. mental arithmetic, calculating percentages, converting measurements, estimating quantities)?"). Ratings had to be given on a ten-point Likert scale (from 1 "never" to 10 "often").

Short description of results of a correlation analysis computed between subjective arithmetic outcomes and demographical variables

Results are described in Figure **S**1. We found that higher age correlated with lower affective anxiety scores. Higher education correlated with a stronger math self-concept. In comparison to females, males indicated lower levels of affective math anxiety, a stronger math self-concept, and a higher frequency of interactions with numbers. Other correlations were not significant (all $p>.05$). In general, lower math anxiety scores correlated with a stronger math self-concept and more positive attitudes toward mathematics.

Figure S1. Coefficients of a Pearson correlation analysis between subjective arithmetic outcomes and demographical variables for the whole sample (N = 134).

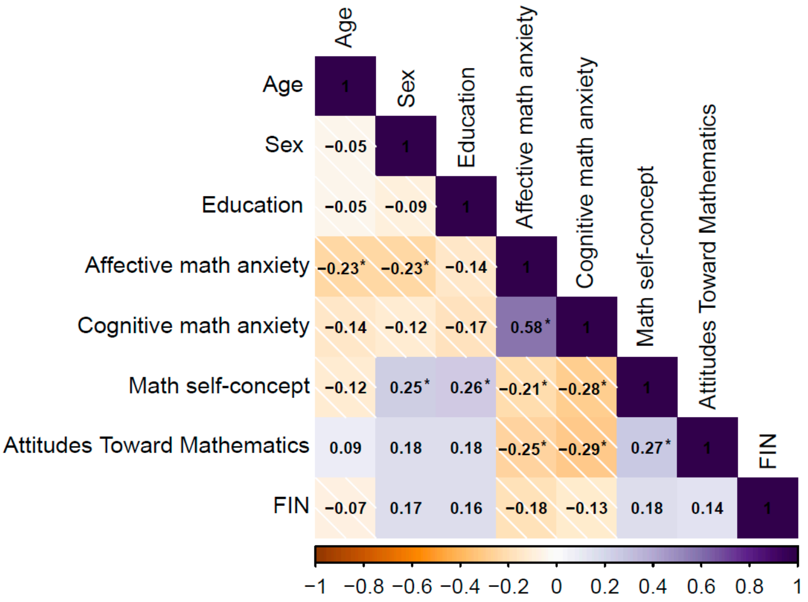

Notes: (*) indicates statistical significance after FDR correction; FIN = Frequency of interactions with numbers.

References

(Aschenbrenner et al., 2000) Aschenbrenner, S., Tucha, O., & Lange, K. W. (2000). Regensburger Wortflüssigkeits-Test (RWT). Hogrefe.

(Bäumler, 1985) Bäumler, G. (1985). Farbe-Wort-Interferenztest (FWIT) nach J. R. Stroop—Handanweisung. Hogrefe.

(Delazer et al., 2003) Delazer, M., Girelli, L., Granà, A., & Domahs, F. (2003). Number processing and calculation—Normative data from healthy adults. The Clinical Neuropsychologist, 17(3), 331–350. https://doi.org/10.1076/clin.17.3.331.18092.

(Folstein et al., 1975) Folstein, M. F., Folstein, S. E., & McHugh, P. R. (1975). Mini-mental state. Journal of Psychiatric Research, 12(3), 189–198. https://doi.org/10.1016/0022-3956(75)90026-6.

(Härting et al., 2000) Härting, C., Markowitsch, H. J., Neufeld, U., Calabrese, P., Deisinger, K., & Kessler, J. (2000). Wechsler gedächtnis test—Revidierte Fassung: Deutsche adaption der revidierten fassung der wechsler-memory scale. Huber.

(Herrmann et al., 1995) Herrmann, C., Buss, U., & Snaith, R. P. (1995). Hospital anxiety and depression scale-german version (HADS-D). Huber.

(Henschel & Roick, 2017) Henschel, S., & Roick, T. (2017). Relationships of mathematics performance, control and value beliefs with cognitive and affective math anxiety. Learning and Individual Differences, 55, 97–107. https://doi.org/10.1016/j.lindif.2017.03.009.

(Lehrl, 2005) Lehrl, S. (2005). Mehrfachwahl-Wortschatz-Intelligenztest: MWT-B (5th ed.). Spitta.

(Petermann et al., 2016) Petermann, F., Jäncke, L., & Waldmann, H.-C. (2016). Neuropsychological Assessment Battery Deutschsprachige Adaptation der Neuropsychological Assessment Battery (NAB) von Robert A. Stern und Travis White. Hogrefe.

(Smith, 1973) Smith, A. (1973). Symbol digit modalities test [Dataset]. APA PsycTests.

(Tapia, 1996) Tapia, M. (1996, November 6). The attitudes toward mathematics instrument. Annual Meeting of the Mid-South Educa-tional Research Association.
